# Identification of DNA Methylation Changes in European Beech Seeds during Desiccation and Storage

**DOI:** 10.3390/ijms24043557

**Published:** 2023-02-10

**Authors:** Marcin Michalak, Beata Patrycja Plitta-Michalak, Jan Suszka, Mirosława Zofia Naskręt-Barciszewska, Szymon Kotlarski, Jan Barciszewski, Paweł Chmielarz

**Affiliations:** 1 Department of Plant Physiology, Genetics and Biotechnology, Faculty of Biology and Biotechnology, University of Warmia and Mazury in Olsztyn, M. Oczapowskiego 1A, 10-721 Olsztyn, Poland; 2 Department of Chemistry, Faculty of Agriculture and Forestry, University of Warmia and Mazury in Olsztyn, Plac Łódzki 4, 10-721 Olsztyn, Poland; 3 Institute of Dendrology, Polish Academy of Sciences, Parkowa 5, 62-035 Kórnik, Poland; 4 Institute of Bioorganic Chemistry, Polish Academy of Sciences, Z. Noskowskiego 12/14, 61-704 Poznań, Poland; 5 NanoBioMedical Centre, Adam Mickiewicz University, Wszechnicy Piastowskiej 3, 61-614 Poznań, Poland

**Keywords:** desiccation, DNA methylation, epigenetics, European beech, intermediate seeds, long-term storage, 5-methylcytosine

## Abstract

Ageing and deterioration of seeds is a major problem for the maintenance of seed quality and viability during long-term storage. Prediction of early stages of seed deterioration in order to point out the plantlets’ regeneration time is a major challenge of successful storage. In preserved seeds, damages accumulate within cells at the rate mainly related to their moisture content and temperature of storage. Current research reveals global alterations in DNA methylation in lipid-rich intermediate seeds during desiccation and storage at various regimes covering nonoptimal and optimal conditions. We show for the first time that monitoring of 5-methylcytosine (m^5^C) level in seeds can be used as a truly universal viability marker regardless of postharvest category of seeds and their composition. For seeds stored up to three years, in varied conditions, moisture content, temperature, and time of storage had significant influence on seedling emergence and DNA methylation (*p* < 0.05). Similarities among lipid-rich intermediate and orthodox seeds regarding different reactions of embryonic axes and cotyledons to desiccation are newly revealed. Along with previous studies on seeds dramatically different in desiccation tolerance (recalcitrant vs. orthodox), results regarding lipid-rich seeds positioned in-between (intermediate) prove that maintaining global DNA methylation status is crucial for maintaining seed viability.

## 1. Introduction

The common beech (*Fagus sylvatica* L.) is one of the most important broadleaved species as it is the most abundant in forests distributed across continental Europe. It is also the most economically important species for Central Europe due to its availability and the diverse usage of the wood. In the past centuries, the total area of beech forests decreased considerably due to human activity; however, in recent decades, an expansion of beech has been reported for most Central European countries [1,2,3,4]. The introduction of common beech has become a key task of current forest managing strategies, and beech was introduced into conifer monocultures in order to convert them into mixture stands that are more resistant to summer drought induced by climate change, devastating effects of windstorms, and bark beetle outbreaks affecting *Picea abies* (L. Karst.) [1,2,3,5,6,7,8].

Common beech produces seeds in highly irregular seed sets with long intervals between good crops lasting 5 to 10 years [9]. It comes from the fact that beech masting exhibits a distance-dependent synchronicity but also a pattern structured by European continental climate regimes as well as being dependent on summer temperature and precipitation in the one to two years before seed production [10] affecting pollen production [11]. Therefore, often unpredictable weather events may affect seed production stronger than climatic, genetic, or environmental factors [10,12]. Thus, is seems clear that to ensure a sufficient number of seedlings for reforestation, seeds must be stored for a long time; hence, optimal protocols for their effective storage are needed [9,13,14].

Successful long-term storage of beech seeds is challenging as they are characterized by poor longevity during preservation under typical conditions applied for orthodox seeds, i.e., 10% of moisture content (MC) and −18 °C. Currently, seeds of common beech are classified as intermediate [15,16,17]. Seeds from this category do not represent either orthodox or recalcitrant type of postharvest behavior; they tolerate desiccation but cannot survive dehydration below those in equilibrium with about 40–50% of relative humidity (RH) and are sensible to subzero temperatures or combined effect of drying and cooling [18,19,20]. Although these seeds were found to remain highly viable during 8 years of storage at 5.4% MC and −8.15 °C (265 K) and are assigned as orthodox [21,22], other investigations presented contradictory results showing beech seeds appointing the optimal storage conditions as 7.8–11% MC at −10 °C to −20 °C [9,16,23]. Moreover, the quality of stored seed lots may vary considerably [9]. During storage, some seed lots quickly lose their initial high viability due to accumulation of cellular damage; thus, more time is necessary for seeds to germinate. Importantly, the true values of germinability can only be assessed after a full stratification and germination test that lasts longer than 20 weeks [9]. However, it is important to notice that a germination test conducted in the laboratory under optimal conditions might not provide real information about the seed deterioration stage; thus, seeds showing few signs of aging might give poor germination results in the more stressful field conditions [13]. Moreover, because of the time consuming stratification-germination procedure, the data on seed quality can be obtained too late to be able to pretreat the seeds for sowing in spring, while cold storage until the following year is associated with a gradual decline in seed quality [9]. An additional problem related to storage is that one of the reasons for seed decay is a fast growth of mold fungi on the surface of beech seeds. The fungi are not visible at the time of collection, but when the relatively moist nuts are temporarily stored after collection (before drying) fungi grow intensively [9]. As a consequence, the evaluation of beech seeds’ viability and assessment of deterioration rate during storage is difficult.

The purpose of this work is to track global DNA methylation changes during desiccation and in varied storage regimes affecting viability of beech seeds in order to observe aging-related changes in methylome and verify the proper conditions for storage ensuring epigenetic stability. It is known that epigenetic mechanisms regulate chromatin structure, gene expression, transposon mobility, and DNA recombination. Epigenetic modifications, including 5-methylcytosine (m^5^C), are subjected to dynamic changes in response to endo- and exogenous stimuli. Studying the changes in plant methylome under stressed conditions has become an essential issue in order to better understand the molecular mechanisms underlying plant stress responses [24,25]. Previously, we showed desiccation and aging related changes in global m^5^C levels in seeds of recalcitrant, orthodox, and short-lived intermediate categories [11,26,27,28,29,30]. Importantly, in our last research entire poplar (*Populus nigra* L.) seeds without separation for embryonic axes and cotyledons were tested. Poplar seeds, contrary to beech seeds, have a low amount of storage lipids and contain developed chloroplasts that may be the main source of oxidative stress leading to the fast aging in those seeds [30,31,32]. The current work shows for the first time the impact of applied treatment (desiccation and storage) on common beech intermediate seed methylome. Significantly, beech seeds are rich in lipids, as the fat content reaches ~28%, and are therefore considered to be so-called oil-seed crops. Based on the fatty acid composition, beech seed oil may be classified as oleic-linoleic acids seeds [17]. Therefore, their composition is significantly different from poplar seeds. Moreover, although there is unclear correlation between seed mortality and content or chemical changes of lipids, they nevertheless continue to be linked to poor storage quality mostly due to interaction between water and crystalized or fluid lipids that might exacerbate damage to imbibing cells [31]. Therefore, we have intended to investigate whether in beech seeds with such characteristics, DNA methylation changes also contribute to their viability loss. The question was also whether beech seeds share the pattern with one of seed postharvest categories or whether they follow their own path.

## 2. Results

### 2.1. The Impact of Desiccation on Seed Viability and Global m^5^C Level

**Figure 1:**
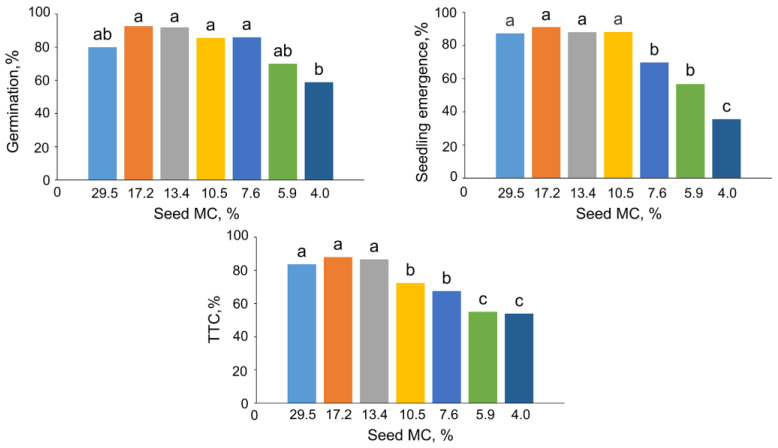
Effect of seed moisture content (MC) on the germination, seedling emergence, and respiratory activity (TTC assay) of *Fagus sylvatica* L. seeds. Values labeled with different letters are significantly different at *p* ≤ 0.05, analysis of variance (ANOVA), Tukey test. Data represent the mean ± se (*n* = 5).

Freshly collected (control) beech seeds with a 29.5% of MC had an initial germination of 80%. Gradual desiccation of these seeds up to an MC of 4% resulted in a reduction of a germination, with the lowest value of 59% (Figure 1). The emergence of seedling of control seeds was 87%, and it declined significantly after desiccation to the MC of 7.6%. After the desiccation to the lowest MC, seeds exhibited a seedling emergence level of 35.5% (Figure 1). According to the TTC staining assay, approximately 84% of the embryos extracted from control seeds were viable. The viability dropped significantly twice after desiccation of seeds up to 10.5% and further to 5.9% of MC (72% and 54% of metabolically active seeds, respectively) (Figure 1).

**Figure 2:**
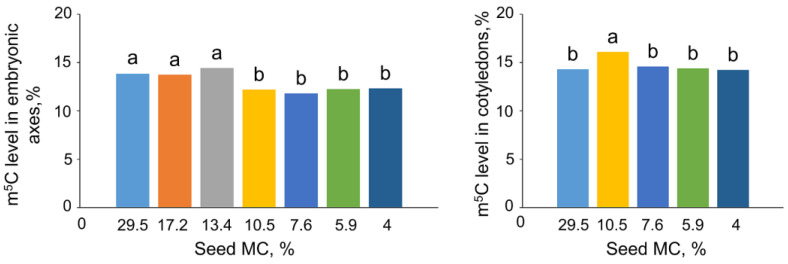
Effect of seed moisture content (MC) on the global DNA methylation of *Fagus sylvatica* L. embryonic axes and cotyledons. Values labeled with different letters are significantly different at *p* ≤ 0.05, ANOVA, Tukey test. Data represent the mean ± se (*n* = 5).

Embryonic axes of control seeds at an MC of 29.5% exhibited a 13.8% of m^5^C, while the m^5^C level in cotyledons was 14.3% (Figure 2). A decrease in the MC of seeds to 10.5% induced a statistically significant decrease in m^5^C to 12% in embryonic axes. Further drying of seeds did not result in decreased m^5^C content. The level of DNA methylation in cotyledons was equal in almost all measured samples (approx. 14.5%); however, at MC of 10.5% the m^5^C level was higher (16.1%). At the same MC, the percentage of m^5^C was higher in cotyledonary tissue than in embryonic axes.

**Figure 3:**
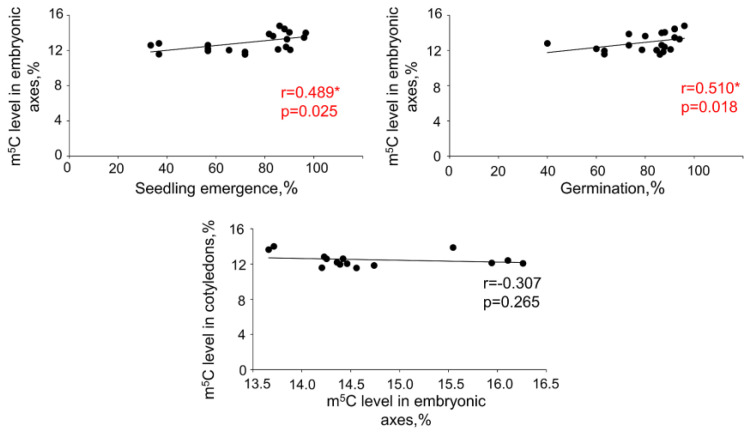
Spearman correlation coefficient (r) between seedling emergence and global DNA methylation level in embryonic axes, germination capacity and global DNA methylation level in embryonic axes, and global DNA methylation level in embryonic axes and cotyledons. Values marked with * are significantly different at *p* < 0.05 and have been additionally marked in red.

**Figure 4:**
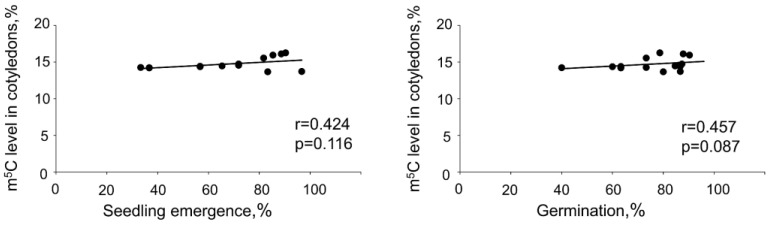
Spearman correlation coefficient analysis (r) between seedling emergence and global DNA methylation level in cotyledons, germination capacity, and global DNA methylation level in cotyledons.

Statistical analyses revealed a positive correlation between germination or seedling emergence and global m^5^C levels in embryonic axes. However, no such correlations were detected when m^5^C amount in cotyledons were taken into consideration (Figure 3 and Figure 4). No relations between DNA methylation level changes in embryonic axes and cotyledons were noticed.

### 2.2. Seed Viability and Global DNA Methylation Changes in Seeds Stored up to 3 Years at Various Regimes

**Figure 5:**
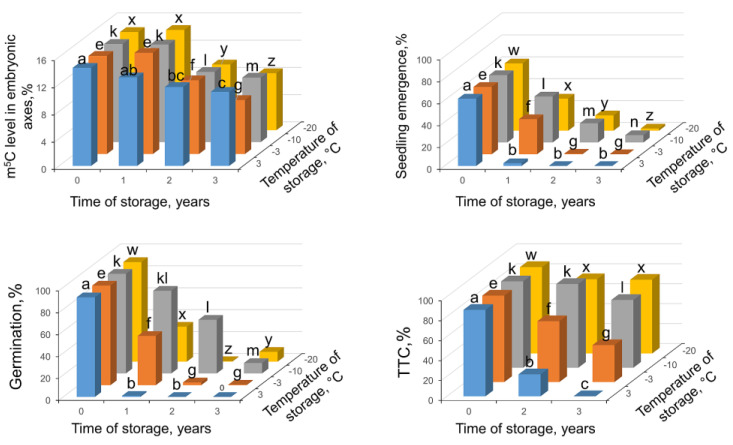
Changes in m^5^C in embryonic axes, seedling emergence, germination capacity, and viability in measured TTC assay in *Fagus sylvatica* L. seeds desiccated to MC of 13.4% and subsequently stored at four various temperatures (3, −3, −10, −20 °C) for one, two, and three years. Values labeled with different letters are significantly different at *p* ≤ 0.05, ANOVA, Tukey test. Data represent the mean ± se (*n* = 5).

Seeds at highest MC of 13.4% germinated at 91% and showed seedling emergence at 61.5%. In Tetrazolium Chloride (TTC) assay, they showed 86% of viability. All viability tests indicated that seeds stored at this MC and at −10 °C demonstrated the highest results of metabolic competence, germinability, and seedling emergence (Figure 5). Nevertheless, after three years of storage, only 7% of seeds produced seedlings (Figure 5). Storage at 3 °C was the most deleterious for these seeds, and after one year of storage their germinability and seedling emergence was residual. When global m^5^C levels were analyzed, it revealed significant decline of DNA methylation level measured after 2 years of storage in all applied storage regimes. Further storage resulted in another significant drop in m^5^C level (Figure 5).

**Figure 6:**
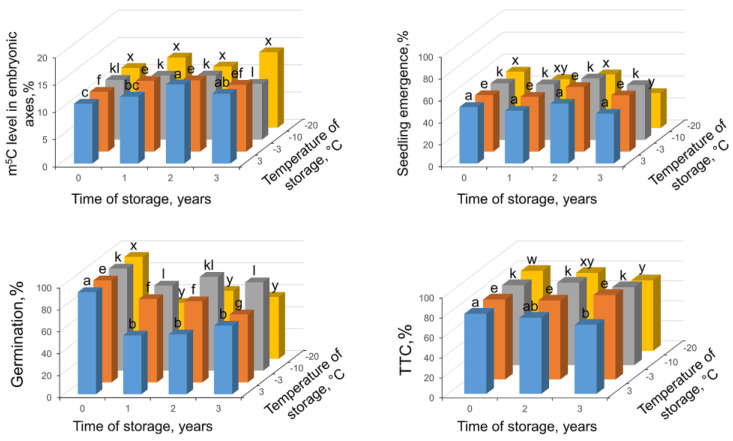
Changes in m^5^C in embryonic axes, seedling emergence, germination capacity, and viability in measured TTC assay in *Fagus sylvatica* L. seeds desiccated to MC of 7.6% and subsequently stored at four various temperatures (3, −3, −10, −20 °C) for one, two, and three years. Values labeled with different letters are significantly different at *p* ≤ 0.05, ANOVA, Tukey test. Data represent the mean ± se (*n* = 5).

Nonstored control seeds at 7.6% of MC showed high germinability (93%); however, only half of seeds produced seedlings (Figure 6). In addition, 80% of these seeds were stained red in TTC assay showing metabolic competence. Storage of seeds at different temperatures for three years caused a significant decrease in germinability after one year (up to 52–78% depending on temperature); however, after further storage in almost all conditions (except the lower germinability after 3-year storage at −3 °C) germinability was on the same level as seedling emergence (Figure 6). TTC assay gave the lowest results only after 3-year storage at 3 °C and −20 °C (69 and 71%, respectively). DNA methylation level in seeds stored at −20 °C and −10 °C was not changed with statistical significance. At the highest temperatures of −3 °C and 3 °C, a significant increase of m^5^C level during storage was observed up to 13 and 14.5% after 2 years followed by decline when measured after another year of storage.

Results obtained for seeds at various MCs stored up to 3 years at four different temperatures indicated that temperature as well as time of storage and moisture content of seeds had significant influence on seedling emergence and DNA methylation level (*p* < 0.05) (Table 1 and Table 2). Moreover, there were significant interactions between all mentioned factors.

## 3. Discussion

Conservation of seeds is the primary method of choice when attempting to preserve the gene pool of a plant species [20,30,33,34,35,36]; therefore, deterioration of seeds during storage has profound influences on agriculture and conservation efforts [37]. Seed aging processes resulting in viability decline and final decrease of plantlet amount are dependent on storage conditions, which are the temperature and RH that determine seed MC. Storage conditions affect various metabolic and biophysical alterations [31,38,39,40]; therefore, longevity of seeds is determined by the general response to storage conditions [37] as well as their genetic properties [41]. There were multiple efforts addressing the subject of poor storability of beech seeds, mostly focused on tracing membrane damage and changes in lipid compositions, as well as activity of reactive oxygen species (ROS) and elements antioxidant system [14,16,41,42]. In addition, a proteomic approach in analyzing the efficiency of stratification process, regulation of this process driven by hormones, and impact of endo- and exogenous polyamines was undertaken [43,44]. However, no previous analyses of DNA methylation changes were made in this context. DNA methylation is an epigenetic process that is involved in regulation of genomic structure and function including transcriptional silencing of transposable elements (TEs), thus in the preservation of genome integrity as well as regulation of gene expression. Still, opposite to the methylation status of a particular gene, a global approach in monitoring genome DNA methylation dynamics creates a link to distinctive physiological states of tissue or plant phenotypes [29,30,45,46]. Indeed, based on DNA methylation variation observed in a natural population of *Arabidopsis thaliana* L., it was shown that differently methylated positions (DMPs) were not proved to have any effect in vivo suggesting that most natural DMPs are functionally inconsequential. A similar observation was made for differently methylated regions (DMRs) located within genes in CG context expected for gene body methylation (gbM). Therefore, it is plausible that the most natural CG-DMRs in genes are functionally inconsequential as well [25,47]. On the other hand, research on seeds showed that genome-wide changes in m^5^C were highly correlated with a loss in viability; therefore, the functional link between global changes in m^5^C and seed deterioration is manifested [27,30,48,49,50,51]. In fact, double mutation of methyltransferase 1 (MET1) and chromomethylase 3 (CMT3) genes had much more dramatic effect on embryogenesis, seed viability, and plant development compared to a mutation in just one gene, which suggests that a plant genome is epigenetically modified in a partially redundant fashion, and DNA methylation must be reduced below a critical threshold level before its role in seed viability is evident [30,48]. Consequently, the presented study aimed to assess changes in global m^5^C level in intermediate lipid-rich beech seeds during storage at different MCs and in various temperatures in relation to their viability. The storage regimes covered optimal conditions providing the highest possible seedling emergence as well conditions expected to cause a decrease in viability. Characterizations of DNA methylation dynamics as a function of temperature, MC, and time aid in understanding of beech seed desiccation sensitivity and deterioration process resulting in their poor storability.

It was previously shown that beech seeds tolerate desiccation in the range of 7.8–11.5% of MC [23,52]. In current research, however, the investigation of desiccation tolerance was extended allowing observation of a decline in viability measured in three different viability tests, i.e., total germination capacity, seedling emergence, and TTC staining during gradual desiccation of seeds from 29.5% up to 4% of MC (Figure 1). Even though significant decline in germination is observed only after desiccation up to 4%, seedling emergence dropped when seeds were desiccated to 7.6%. Such a result confirms that seedling emergence is a better measure of seed viability since it represents the actual number of regenerated plants. Seeds showing high germination but poor emergence are considered low vigor seeds having poor storage potential [13,53]. Significantly, TTC assay revealing desiccation induced a decline in respiratory activity at 10.5% of MC, which might be considered as a first sign representative of biochemical lesions [54]. Moreover, when DNA methylation level was analyzed, the sudden decline was noticed in embryonic axes desiccated to this MC, and it remained constant in further desiccation steps. Previously, we showed that viability decline of orthodox and recalcitrant seeds subjected to desiccation [29,55] is highly correlated with decline of DNA methylation level in embryonic axes isolated from seeds of these categories. Hypomethylation-related viability decline in *A. thaliana* tissues was also reported [50]. To the contrary, when the m^5^C amount was increasing, the viability of seeds remained high and constant as in case of orthodox *Pyrus communis* L. seeds and *Populus nigra* L. intermediate short-lived chlorophyllous seeds that withstand desiccation [26,30,56]. This suggests that the higher DNA methylation is beneficial for seeds [30,56,57]. Based on these observations altogether, it can be stated that measurement of global DNA methylation level and its comparison to the initial methylation state of genomes showing the increasing or decreasing tendency can possibly give an indication as to viability status of a seed lot regardless of the postharvest category they are assigned and also irrespective of seed composition (lipid-rich seeds vs. seeds with chlorophyllous cells). Importantly, each seed lot may differ in MC, mass accumulation, and consequently maturity, which may impact m^5^C. Indeed, it was shown that the proportion of methylated DNA was changing with seed mass accumulation [57]. Therefore, the relative changes in m^5^C level rather than the absolute value are the most relevant for comparison. Here, we observe that in intermediate lipid-rich seeds the decrease in m^5^C is correlated with viability decline (Figure 3). This confirms that deterioration of seeds can be indicated with DNA methylation status without prior information about postharvest physiology, desiccation tolerance, and seed composition.

Opposite to embryonic axes, the amount of m^5^C in cotyledons was constant after desiccation of beech seeds, but at 10.5% of MC an upsurge was observed. We showed preciously that cotyledons of orthodox *Acer platanoides* L. seeds did not show any changes in DNA methylation level after severe desiccation, whereas in recalcitrant seeds of *Acer pseudoplatanus* L. a statistically significant drop of m^5^C related to viability decline was observed, which can be considered as a sign of recalcitrance [29]. Here, the lack of DNA methylation changes in cotyledons is similar to orthodox-type seeds, although the intriguing nature of the observed increase is currently unidentified for us and needs further explanations. Nevertheless, the observed m^5^C changes during desiccation can be considered as tissue specific in seeds of all three postharvest categories. That specificity of reaction of seed tissues to desiccation is expected to come from the fact that embryonic axes of seeds are characterized by greater water sorption and contain more water than cotyledons at the same RH. Such difference between both tissues in water sorption is considered to have a great importance for seed viability and possibly contribute to higher sensitivity of embryonic axes to storage conditions [16] that plausibly emanate as a hypomethylation of tissue. The molecular mobility is expected to be higher in embryonic axes, leading to disruption of metabolic balance and possibly affecting DNA methylation status as a consequence. Indeed, also in our experiment the level of MC was higher in embryonic axes at the time of collection and during desiccation (Table S1), and DNA methylation changes that correlated with seed deterioration were observed only for embryonic axes. Moreover, we perceive the link between imbalance in cellular redox environment of beech seeds and ROS-related effect on DNA methylation [29,55,58]. It was previously shown that embryonic axes of beech are exposed to a greater degree to detrimental chemical reactions leading to oxidative stress and ROS overproduction and consequently to the multiple and diverse damages to metabolism and cellular structures [16,41]. Moreover, participation of ROS in DNA oxidation and fragmentation was previously showed in recalcitrant embryos [55,59]. In the case of intermediate seeds, beech embryos were also analyzed in terms of ROS accumulation showing their markedly higher level in embryonic axes than in cotyledons during storage at 7–8% of MC and at −10 °C [42]. This observation is in concordance with anticipated ROS-related m^5^C decline in embryonic tissue.

Longevity of beech seeds declines during storage even under optimal conditions, giving the reduction of germinability up to 20% and 14% after 10 or 13 years, respectively [14,42]. Poor storability of these seeds elicited multiple efforts exploring its biochemical and physiological causes. Here we observed viability and global DNA methylation in optimal storage conditions of MC and temperature as well as nonoptimal in order to detect gradual decrease in viability in the period of 3 years. At both chosen MCs (7.6 and 13.4%), the initial viability of beech seeds was above the minimum 80% recommended for long-term storage [60]. As the temperature is one of the most important environmental factors regulating the percentage and rate of seed germination [61], we chose optimal storage temperatures of −20 °C and −10 °C as well as higher ones (−3 °C and 3 °C). The rationale for that approach was that degradative reactions are controlled by chemical potential of water and its availability for chemical reactions and that the optimal MC changes with temperature [16]. The combination of applied conditions allowed us to observe progressive decline in seed viability. Higher temperature of storage would cause much rapid decline of viability, while our intention was to capture the changes during storage in years. Moreover, beech seeds can be infected by microflora (fungi and bacteria) during storage, and the quality of seeds gradually declines as a result of their activity. Accelerated aging, usually induced by storage at 25–35 °C, stimulates the seed microflora to intensive growth and activity [9]. Therefore, to observe the aging process over a prolonged time related to immanent seeds’ aging processes we chose the temperatures far from these typically used for accelerating aging. Additionally, we wanted to avoid the falsification of TTC assay resulting from microflora activity [9].

At the MC of 13% (Figure 5), previously reported to be above optimal for storage, all viability tests showed progressive deterioration of seeds during storage up to 3 years in all temperature conditions. Storage at the highest chosen temperatures was deleterious for seeds, and even though after 2 and 3 years seeds remain metabolically active, germination and seedling emergence was completely lost, and a decrease of DNA methylation level in embryonic axes was detected. It can be ratiocinated that hypomethylation results from active metabolism, either enzymatic activity or ROS related damages [55,58]. Indeed, TTC results revealed that seeds were metabolically active during storage at lower temperatures. Moreover, our results are in agreement with the previous study [60] showing that storage at −5 °C of seeds at MC of 13% for 4 years almost completely diminished the viability, however, with observed sudden decrease of viability in few months before testing. Seeds after 5 years were completely dead. It was also reported that for beech seeds at MC of 15% and higher, cell structure is destroyed due to water crystallization at low temperatures [16]. Current research proves that not only physical damages to beech seeds resulting from ice nucleation contribute to viability decline [16] but also biochemical processes as DNA demethylation.

At the MC of 7% and temperature of −10 °C, the germinability of seeds was the highest; however, the seedling emergence after 3 years was between 32% for −20 °C and 50–51% for −3 and −10 °C, respectively (Figure 6). Such storage regimes (MC of 7%, −10 °C) were assigned as optimal for beech seeds [14,23,42]; however, only germination as protrusion of radicle was estimated. Breeding of seedlings for 7–10 weeks gave close results for all conditions and all test times. When DNA methylation was measured, only at 3 °C was a significant increase in relation to control seeds observed while in other storage temperatures we noticed insignificant changes or an increase (−3 °C); however, this was followed by decline after 3 years leading to insignificant results in relation to control. Previously, it was shown that orthodox seeds of constant viability sustain global DNA methylation levels [26,56]. This is true also for lipid-rich intermediate seeds tested in current research, which revealed orthodox-type behavior when DNA methylation dynamics were analyzed during storage at optimal conditions. Such observation showing unaffected seedling emergence and constant or increasing level of m^5^C is congruent with our description of m^5^C status as a viability marker.

## 4. Materials and Methods

### 4.1. Plant Material, Assessment of Water Content, and Desiccation of Seeds

Seeds of *Fagus sylvatica* L. (European beech) were collected from the population in Gryfino, Poland (N53° 14′ 44″, E14° 32′ 47). The moisture content (MC) of seeds was calculated on a fresh weight basis using a previously described formula [26]. Freshly collected seeds had MC at the level of 29.5%. Then samples of seeds were dried on a laboratory bench at 20 °C to a 7.6% of MC that lasted up to eight days. In order to obtain MC values lower than 7.6%, seeds were placed in a drying box on blotting paper and desiccated over silica gel. Finally, beech seeds were desiccated to six levels of MC (17.2, 13.4, 10.5, 7.6, 5.9, and 4.0%). The duration of the desiccation ranged from several days to two–six weeks in the case of the lowest MC. Moisture contents of seeds were assessed by drying at 103 °C ± 2 °C for 24 h.

### 4.2. Seed Storage Conditions

Seeds of *F. sylvatica* were stored at two MCs: 13.4% and 7.2% in four different temperature conditions (3, −3, −10, and −20 °C) for up three years. Seeds were packed in tightly closed polyethylene bags during storage.

### 4.3. Viability Assessment

Prior to germination tests, stratification of dormant beech seeds was required after desiccation. Seeds were placed in a substrate consisting of a moist mixture (1:1, *v*/*v*) of quartz sand (<1 mm fraction) and sieved peat (pH 3.5–4.5). Seeds, mixed with the substrate (1:3, *v*/*v*), were placed in 0.25 l plastic bottles, until the first germinated seeds (<5%), defined as seeds with a 2–3 mm long radical, were observed. This was used as a visible indicator that the seeds were released from dormancy. Water was added to the substrate as needed to keep it moist, and the seeds were monitored for fungal infections throughout the stratification treatment. *F. sylvatica* seeds required 8–10 weeks at 3 °C to complete stratification.

Germination and seedling emergence tests were conducted on separate samples of seeds, using four replicates of 50 seeds each. Seed germination tests were performed in the dark at 3 °C, after 5% of seeds showed signs of radicle protrusion, in a mixture of sand with peat, similar to that used for the stratification. Each bottle containing seeds was closed with a lid that had several holes 5 mm in diameter, which enabled gas exchange and protected the substrate against excessive drying. The condition of the seeds and the substrate was monitored every week. The number of germinated seeds was counted each week, and at that time the water in the substrate was replenished. *F. sylvatica* seeds required 10–11 weeks at 3 °C to complete the germination test.

Seedling emergence assays were conducted after the completion of stratification by transferring them to a cyclically alternating temperature (20 °C/3 °C for 8 h/16 h, light/dark photoperiod) in a mixture of sand with peat, similar to that used for the stratification and germination tests. After stratification, seeds were sown in plastic boxes containing the substrate at a depth of 10 mm and covered with a layer of sand. The boxes were covered with a transparent lid (allowing penetration of light) to ensure that adequate moisture was maintained. Seedling emergence tests, such as germination tests, were conducted using constant temperature of 3 °C, until seedlings were ca. 20–30 mm in height. Boxes (without the lid) with the seedlings were then moved into the light (60 μmol m^−2^ s^−1^ for 16 h a day) at 25 °C. *F. sylvatica* seeds required 18–20 weeks at 3 °C to complete the seedling emergence test.

A Tetrazolium Chloride (TTC) assay was conducted according to the International Rules for Seed Testing [62]. The reduction of colorless 2,3,5 triphenyl tetrazolium chloride (TTC) into insoluble pink/red triphenyl formazan was taken as a sigh of respiratory competence. The assumption is that TTC is reduced by components of the mitochondrial electron transport chain [54]. Embryos were soaked in a solution of 1% TTC. The test was carried out in 50 mL covered vessels containing 25 mL aqueous sterile solutions of 1% TTC. The TTC test was conducted on four biological replicates, each with 30 seeds. Seeds were incubated in the TTC solution in the dark at 30 ± 1 °C for 24 h. Embryos that remained white (unstained) or had unstained areas close to embryonic axes that exhibited evidence of staining were considered as dead seeds, while embryos that were stained pink to red were classified as living.

### 4.4. DNA Isolation and Assessment of Global m^5^C Levels

Total genomic DNA was separately extracted from embryonic axes and cotyledons with a Qiagen DNAeasy Plant Mini Kit^TM^ (Qiagen, Hilden, Germany). Each assay consisted of five biological replicates, which comprised either five embryonic axes or three cotyledons. A TLC-based method was used for the chromatographic separation of m^5^C from other DNA bases, as well as RNA contamination, since it provides very precise separation. All nucleobases were labeled with radioactive phosphate to enable a highly sensitive determination of the level of genome-wide methylation in DNA samples derived from plant tissues of limited size, such as the embryonic axes used in this study. Analysis of the global level of m^5^C in DNA of seeds was carried out and calculated as previously described [63]. Dried DNA (1 µg) was digested to completion (6 h) with 0.001 U of spleen phosphodiesterase II and 0.02 U of microccocal nuclease in 20 mM succinate buffer containing 10 mM CaCl_2_ at 37 °C. The resulting hydrolysate (0.3 µg) was then labelled with 1 µCi [γ-^32^P] ATP (6000 Ci mmol^−1^ Hartmann Analytic, Braunschweig, Germany) and 1.5 U of T4 polynucleotide kinase in 10 mM bicine-NaOH buffer (pH 9.7) containing 10 mM MgCl_2_, 10 mM DTT, and 1 mM spermidine. After incubation for 30 min at 37 °C, 0.03 U of apyrase in 10 mM bicine-NaOH buffer was added, and the mixture was incubated for 30 min. Subsequently, 0.2 µg of RNase P1 in 500 mM ammonium acetate buffer pH 4.5 was used for 3′phosphate cleavage. Analysis of [γ-^32^P] m^5^C was performed with 2D TLC on cellulose plates (Merck) in isobutyric acid/NH_4_OH/H_2_O (66/1/17), (first direction) and 0.1 M sodium phosphate pH 6.8—ammonium sulfate- *n*-propanol (100 mL/60 g/1.5 mL), (second direction). Radioactivity was measured with a FLA-5100 Fluoro Image Analyzer and Multi Gauge 3.0 Software. The image analysis procedure used to quantify the level of m^5^C accounts for the level of both cytosine (C) and m^5^C. The level of m^5^C in each biological replicate was measured five times. The R ratio was calculated using the following formula:

R (%) = m^5^C/m^5^C + C × 100 (1)

### 4.5. Statistical Analysis

STATISTICA version 11.0 (StatSoft, Tulsa, OK, USA) and JMP 12 (SAS Institute Inc., Cary, NC, USA) software was used for the statistical analyses. All percentage data were *arc-sin*-transformed prior to analysis according to the Bliss equation [64]. In all figures, however, nontransformed data are presented to simplify the interpretation of biological relevance. Two-way analysis of variance (ANOVA) was used to determine the significance of the effect of storage time, moisture content, and storage temperature on seedling emergence and global DNA methylation level of embryonic axes. Tukey test was used to determine significant differences between sample means at *p* ≤ 0.05. Separate ANOVAs and post-hoc tests were performed on data for germination, seedling emergence, the TTC assay, global DNA methylation level of embryonic axes, and global DNA methylation level of cotyledons. The correlations were tested using the Spearman correlation coefficient analysis. Error bars indicate standard errors (s.e.) of the mean within an individual treatment.

## 5. Conclusions

When seeds are stored, damage accumulates within a cell at the rate dependent on MC and temperature, and the process goes on until the seed loses viability and finally dies. A critical factor that has a major economic impact is beech seed quality during storage, and its decline that can occur from year to year. Therefore, reliable up-to-date information about the quality of stored seeds is needed to indicate the time when seeds need to be delivered from warehouse to nurseries. Additionally, regarding the fact of inevitable aging of seeds, the preserved seed lots may vary considerably [9,13], and even if the optimal beech seed storage protocol is applied, there are seed lots that lose their high initial viability and deteriorate after few year of storage, whereas others retain their high viability. Measurement of the global DNA methylation level could be successfully used to predict potential viability of seeds of orthodox, recalcitrant, and intermediate categories regardless their composition (e.g., lipid-rich beech seeds vs. poplar seeds with active chloroplasts). Measurement of global m^5^C changes allows differentiation between less vigorous seeds and vigorous ones without excessive consuming of seed samples. Here, we proved that optimal for storage seed MC of 7.6% relates to global DNA methylation stability contrary to higher nonoptimal MC. However, the common phenomenon of genomic hypomethylation in three categories [27,30,56] of seeds confirms that epigenetic regulation strongly affects plant cell metabolism, and exceeding a particular DNA methylation threshold gives an evident sign of physiological failure as decrease of vigor and viability.

## Figures and Tables

**Table 1:** Analysis of variance (ANOVA) of the effect of storage time (0, 1, 2, 3 years), moisture content (7, 13%), and temperature of storage (3, −3, −10, −20 °C) on total m^5^C level of embryonic axes of *Fagus sylvatica* L. ST, storage time; STE, storage temperature; MC, moisture content.

Source Variance	DF	F	*p*
ST	3	119.5	<0.0001
MC	1	7.3	0.0087
STE	3	16.2	<0.0001
MC * STE	3	5.9	0.0012
MC * STE	3	90.6	<0.0001
ST * STE	9	12.7	<0.0001
ST * MC * STE	9	3.3	0.0024

**Table 2:** Analysis of variance (ANOVA) of the effect of storage time (0, 1, 2, 3 years), moisture content (7, 13%), and temperature of storage (3, −3, −10, −20 °C) on seedling emergence *Fagus sylvatica* L. seeds. ST, storage time; STE, storage temperature; MC, moisture content.

Source Variance	DF	F	*p*
ST	3	29.05	<0.0001
MC	1	671.52	<0.0001
STE	3	598.91	<0.0001
MC * STE	3	34.52	<0.0001
MC *STE	3	296.25	<0.0001
ST * STE	9	7.33	<0.0001
ST * MC * STE	9	8.14	<0.0001

## Data Availability

The data presented in this study are available on request from the corresponding authors.

## Supplementary Materials

The following supporting information can be downloaded at: https://www.mdpi.com/article/10.3390/ijms24043557/s1; Table S1: Mean moisture content (%) of seeds, cotyledons, and embryonic axes of *Fagus sylvatica* L. (± standard error), (*n* = 3).
