# Supplementary material for: Identification of DNA Methylation Changes in European Beech Seeds during Desiccation and Storage
Source: Int J Mol Sci. 2023 Feb 10;24(4):3557. doi: 10.3390/ijms24043557 (PMC9968092; doi:10.3390/ijms24043557)
Supplement: Supplementary file 1 [file ijms-24-03557-s001.zip › ijms-2187911-supplementary.pdf]

Table S1. Mean moisture content (%) of seeds, cotyledons, and embryonic axes of *Fagus sylvatica* L. (± standard error), (n=3).

Seed	Cotyledon	Embryonic axes
29.5±0.6	29.95±0.8	46.3±1.2
17.2±0.1	-	-
13.4±0.1	-	-
10.5±0.1	8.3±0.2	11.0±0.1
7.6±0.1	6.8±0.1	8.4±0.1
5.9±0.1	4.9±0.1	5.9±0.2
4.0±0.1	3.5±0.1	5.2±0.1
